# Biogenic vs. Chemical AgNPs: A Comparison of Antimicrobial Potency and Stability

**DOI:** 10.3390/ijms27010062

**Published:** 2025-12-20

**Authors:** Mukil Madhusudanan, Ivan Mijakovic, Priyanka Singh

**Affiliations:** 1 The Novo Nordisk Foundation Center for Biosustainability, Technical University of Denmark, DK-2800 Kongens Lyngby, Denmark; 2 Systems and Synthetic Biology Division, Department of Life Sciences, Chalmers University of Technology, SE-412 96 Gothenburg, Sweden

**Keywords:** silver nanoparticles, green synthesis, plant extract, bacterial supernatant, citrate, antimicrobial, biofilm eradication

## Abstract

This study presents a comprehensive evaluation of the antimicrobial activities of silver nanoparticles (AgNPs) synthesized using three distinct methods: plant extracts, bacterial supernatant, and a conventional chemical method. AgNPs were synthesized from *Crassula ovata* (Jade) leaf extract, *Bacillus licheniformis* bacterial supernatant, and a standard chemical reduction method using trisodium citrate. The synthesized AgNPs were characterized using UV–Vis spectroscopy, Transmission Electron Microscopy (TEM), and Dynamic Light Scattering (DLS). The antimicrobial efficacy of the AgNPs was tested against four pathogenic microorganisms: *Escherichia coli*, *Pseudomonas aeruginosa*, *Staphylococcus epidermidis*, and Methicillin-resistant *Staphylococcus aureus* (MRSA). Our findings reveal significant differences in the biological activities of the AgNPs depending on the synthesis method. The MBC values for the plant extract-synthesized AgNPs were 10 µg/mL for *E. coli*, 12.5 µg/mL for *P. aeruginosa*, 10 µg/mL for *S. epidermidis*, and 15 µg/mL for MRSA. The bacterial supernatant-synthesized AgNPs showed MBC values of 10 µg/mL for *E. coli*, 12.5 µg/mL for *P. aeruginosa*, 7.5 µg/mL for *S. epidermidis*, and 12.5 µg/mL for MRSA. In contrast, citrate-reduced AgNPs exhibited higher MBCs: 60 µg/mL for *E. coli* and *P. aeruginosa*, 40 µg/mL for *S. epidermidis*, and 80 µg/mL for MRSA. Notably, the AgNPs synthesized using plant and bacterial supernatant demonstrated superior antimicrobial activity compared to those synthesized chemically. This comparative study highlights the potential of eco-friendly synthesis routes for producing AgNPs with enhanced biological activities. The findings suggest that plant extract and bacterial supernatant-mediated synthesis of AgNPs could serve as a viable and sustainable alternative to conventional chemical methods, offering promising applications in medical and pharmaceutical fields.

## 1. Introduction

Antimicrobial resistance (AMR) has emerged as a critical global health challenge, threatening the very foundation of modern medicine [1]. The alarming rise in multidrug-resistant pathogens has rendered many of our existing antibiotics ineffective, leading to prolonged illnesses, higher healthcare costs, and increased mortality rates [2]. As the prevalence of these resistant organisms continues to grow, the urgency for developing novel antimicrobial agents has never been greater. The World Health Organization (WHO) has recognized AMR as one of the top ten global public health threats facing humanity, underscoring the severity of the crisis [3].

The rapid spread of resistant microorganisms, including bacteria, viruses, fungi, and parasites, is driven by several factors. These include the misuse of antibiotics in medicine and agriculture, poor infection-prevention practices, and limited investment in new antimicrobial drugs [4]. These factors have accelerated the evolution of resistant strains, creating a vicious cycle in which the development of new drugs struggles to keep pace with the emergence of resistance. As a result, infections that were once easily treatable are now more difficult to manage, leading to longer hospital stays, higher medical costs, and increased mortality. Despite the critical need for new antimicrobial agents, the development pipeline for novel antibiotics has been alarmingly sparse [2]. Consequently, there is a pressing need for innovative approaches to discover and develop new antimicrobial agents capable of effectively combating resistant pathogens.

Nanotechnology offers a promising avenue to address this challenge, with AgNPs emerging as particularly potent candidates. AgNPs have gained significant attention due to their unique physicochemical properties and broad-spectrum antimicrobial activity against a wide range of microorganisms, including antibiotic-resistant strains [5]. These nanoparticles offer a promising alternative to traditional antibiotics. Their small size, large surface area, and high reactivity enhance interactions with microbial cells and improve antimicrobial efficacy [6]. AgNPs exert their antimicrobial effects through multiple mechanisms, including the generation of reactive oxygen species (ROS), disruption of microbial cell membranes, interference with DNA replication, and inhibition of essential enzyme functions [7]. These multifaceted modes of action make it difficult for microorganisms to develop resistance to AgNPs, positioning them as a valuable tool in the fight against AMR. Research has demonstrated that AgNPs can be synthesized using various methods, including chemical reduction, physical methods, and biological approaches. Each synthesis method influences the size, shape, and surface properties of the nanoparticles, which subsequently influence their antimicrobial potency [8].

Silver nanoparticles exhibit antimicrobial activity through several well-established mechanisms. They can generate ROS, which causes oxidative damage to cellular components. AgNPs also disrupt bacterial cell membranes by attaching to the membrane surface and increasing permeability, leading to leakage of intracellular contents [9]. Released silver ions further interact with thiol groups in proteins and enzymes, impairing essential metabolic processes and inhibiting DNA replication [10]. Together, these mechanisms result in strong bacteriostatic and bactericidal effects, against antibiotic-resistant pathogens. Biologically synthesized AgNPs often possess organic capping layers that enhance their interaction with microbial surfaces, which may further contribute to their antimicrobial potency [11].

However, while AgNPs present a powerful tool in the fight against AMR, the conventional chemical synthesis methods used to produce these nanoparticles often raise environmental and health concerns. Chemical synthesis often requires toxic reducing agents and produces hazardous waste. This creates risks for human health and the environment and shows the need for more sustainable production methods [6]. In response to these challenges, the concept of green synthesis of AgNPs has emerged as a sustainable alternative. This approach utilizes biological entities such as plant extracts, bacteria, fungi, and algae as reducing and stabilizing agents, eliminating the need for hazardous chemicals. Green synthesis reduces the environmental and health risks associated with chemical methods and leverages biological systems’ natural reducing power to produce nanoparticles with tailored properties [12].

Several natural biopolymers have also been widely explored for the green synthesis of AgNPs. Materials such as cellulose, chitin, and chitosan possess abundant functional groups that can act as reducing and stabilizing agents, enabling the formation of nanoparticles without the need for chemical reagents. These biopolymers provide excellent biocompatibility and form stable capping layers that enhance nanoparticle stability and antimicrobial performance. Recent studies have demonstrated their effectiveness in producing uniform and functional AgNPs, further highlighting their potential for sustainable nanomaterial synthesis [13,14,15].

*Crassula ovata*, commonly known as the Jade plant, holds significant biomedical potential due to its rich phytochemical profile, which includes compounds such as flavonoids, tannins, and phenolic acids. Traditionally used in folk medicine, the extracts from Jade plant leaves are used to treat a variety of ailments, including wounds, skin warts, and stomach-related issues [16]. Additionally, extracts from the Jade plant have demonstrated a range of pharmacological activities, including anti-inflammatory, antimicrobial, anticancer, and antioxidant effects, highlighting its versatility as a therapeutic agent [17,18]. In the present study, we used the leaf extract of the Jade plant for the synthesis of AgNPs.

Similarly, the soil bacterium *B. licheniformis* is of significant biomedical importance due to its ability to produce a wide range of bioactive compounds, including enzymes like proteases and amylases and antimicrobial substances such as bacitracin [19]. These properties make it valuable in pharmaceutical and industrial applications. Additionally, *B. licheniformis* is recognized for its probiotic potential, contributing to gut health and immune system modulation [20]. Its capacity to synthesize AgNPs further enhances its relevance for innovative antimicrobial therapies. We have used the extracellular synthesis method by using *B. licheniformis* supernatant to convert silver ions into AgNPs. We used the most traditional method of making silver nanoparticles for chemical synthesis and compared them with green synthesis. Thus, the study aims to evaluate and compare the antimicrobial activities of AgNPs synthesized through plant extracts, bacterial supernatant, and chemical methods. This research seeks to determine the most effective synthesis approach for producing AgNPs with superior biological activities by conducting a comprehensive analysis. The findings of this study could provide valuable insights into optimizing the synthesis processes for AgNPs, ultimately enhancing their application in medical and pharmaceutical fields.

## 2. Results

### 2.1. Synthesis and Visual Observation of AgNPs

**Figure 1:**
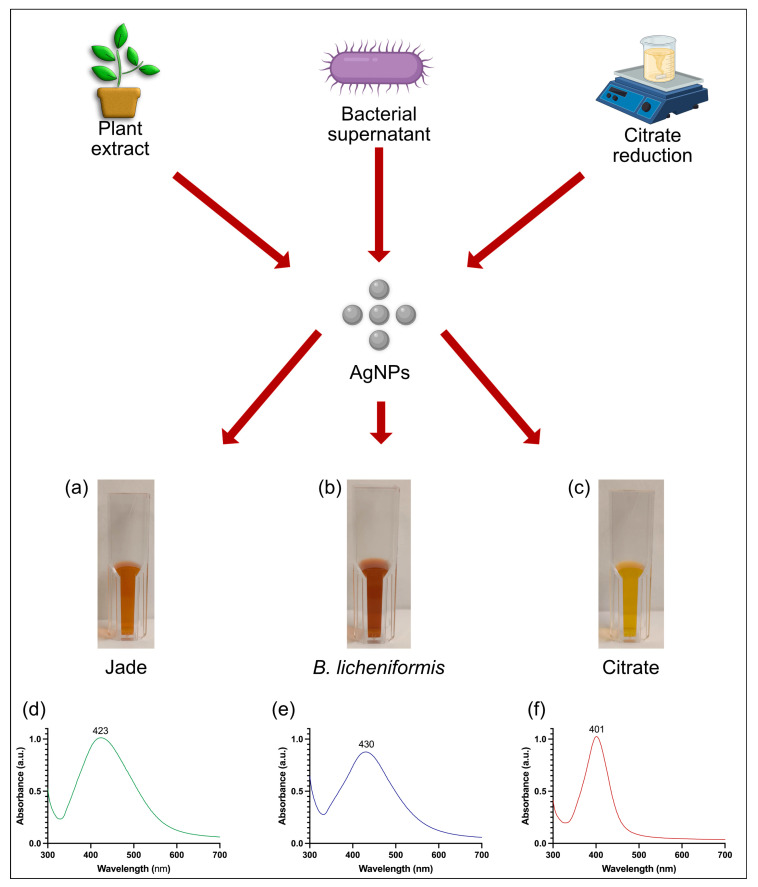
Biological and chemical synthesis of AgNPs using *C. ovata* extract, *B. licheniformis* supernatant, and citrate reduction. Visible and UV-vis spectral examination of AgNPs. Visible picture (**a**) Jade AgNPs, (**b**) *B. licheniformis* AgNPs, (**c**) citrate AgNPs. UV-vis spectra (**d**) Jade AgNPs, (**e**) *B. licheniformis* AgNPs, (**f**) citrate AgNPs.

AgNPs were successfully synthesized using three methods: plant extract-mediated synthesis, bacterial supernatant-mediated synthesis, and chemical synthesis via citrate reduction. For the plant-based synthesis, the reaction parameters were optimized by varying the concentration of AgNO_3_, the ratio of plant extract to water, and the reaction time. Optimal conditions were found to be a 1:5 ratio of extract/water, 3 mM AgNO_3_, and two hours of incubation at room temperature (RT). For bacterial synthesis, the silver salt concentration, temperature (RT & 37 °C), and reaction time were optimized with incubation on a rotary mixer for 1 h at RT with 5 mM of silver salt, giving the best results. Chemical synthesis was carried out as reported by Bastús et al. [21], resulting in the instantaneous formation of AgNPs. Visual inspection of the reaction mixtures revealed color changes from yellowish to brown, indicative of nanoparticle formation in the synthesis medium (Figure 1a–c). The plant extract-mediated synthesis resulted in a brownish-orange color, the bacterial supernatant-mediated synthesis produced a darker brown color, and the citrate reduction method yielded a characteristic yellow color. These color changes suggested the formation of AgNPs due to the surface plasmon resonance (SPR) of the nanoparticles [22].

### 2.2. UV-Vis Spectroscopy Analysis

The formation and optical properties of the AgNPs were confirmed by UV-Vis spectroscopy. The UV-Vis spectra of the AgNPs synthesized by plant extract showed a prominent absorption peak at 423 nm, indicating the presence of nanoparticles with a narrow size distribution. The bacterial supernatant-mediated synthesis produced a similar SPR peak at around 430 nm, suggesting a comparable nanoparticle size [23]. In contrast, the AgNPs synthesized by citrate reduction exhibited a narrow peak at 401 nm, indicating slightly smaller and more homogenous nanoparticles compared to those synthesized biologically (Figure 1d–f). The sharpness and intensity of these peaks further confirmed the successful synthesis of AgNPs by all three methods.

### 2.3. DLS for Particle Size and Zeta Potential Analysis

**Figure 2:**
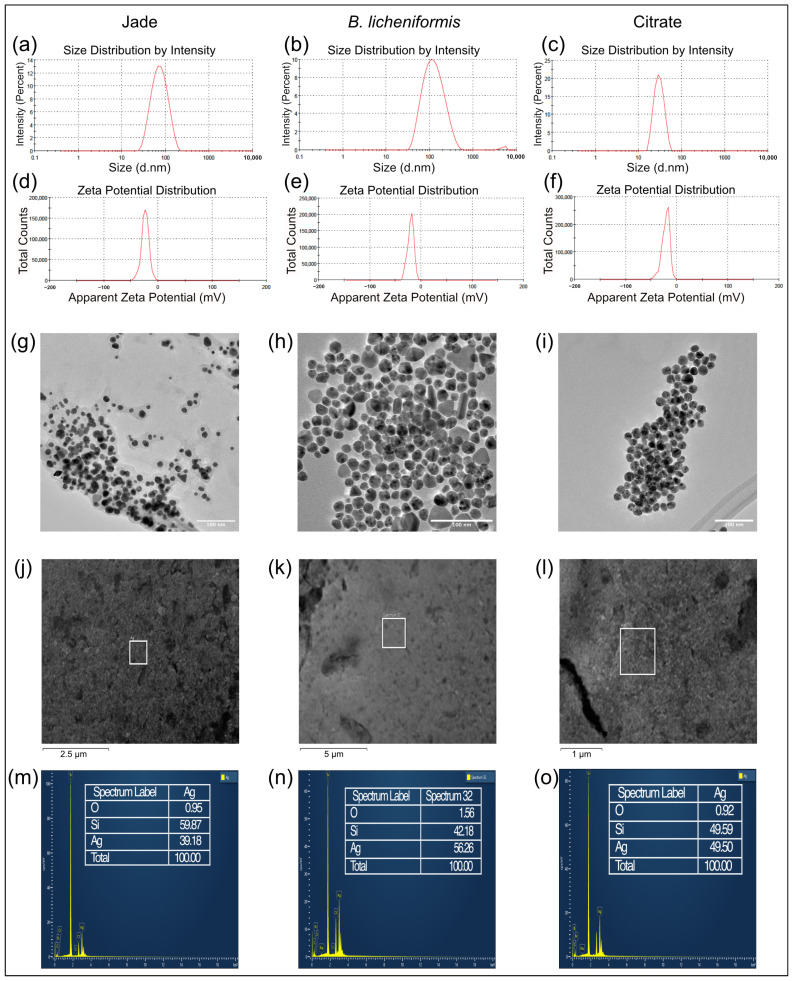
Dynamic light scattering for particle size distribution by intensity (**a**) Jade AgNPs, (**b**) *B. licheniformis* AgNPs, (**c**) citrate AgNPs. Zeta potential measurement (**d**) Jade AgNPs, (**e**) *B. licheniformis* AgNPs, (**f**) citrate AgNPs. TEM micrograph of (**g**) Jade AgNPs, (**h**) *B. licheniformis* AgNPs, (**i**) citrate AgNPs. SEM micrograph with EDX spectra of (**j**,**m**) Jade AgNPs, (**k**,**n**) *B. licheniformis* AgNPs, (**l**,**o**) citrate AgNPs.

DLS was employed to determine the hydrodynamic diameter and size distribution of the synthesized AgNPs. The AgNPs synthesized using plant extract had an average hydrodynamic diameter of 61.28 nm with a polydispersity index (PDI) of 0.217 (Figure 2a), indicating a moderately uniform size distribution. The bacterial supernatant-mediated AgNPs exhibited a slightly larger average diameter of around 108.3 nm with a PDI of 0.23, suggesting a broader size distribution (Figure 2b). The citrate-reduced AgNPs were the smallest, with an average diameter of 28.05 nm and a PDI of 0.073, indicating a highly uniform nanoparticle population (Figure 2c). The elevated Z-average may result from nanoparticles with diverse shapes and sizes because intensity-based DLS measurements are strongly influenced by particle morphology. Larger and irregularly shaped nanoparticles scatter light more intensely, skewing the average size upwards and leading to a higher Z-average [24].

Zeta potential measurements were conducted to assess the stability of the AgNPs suspensions. A negative zeta potential suggests that the nanoparticles are stabilized by electrostatic repulsion. The zeta potential of the plant extract-synthesized AgNPs was found to be −23.8 mV (Figure 2d), while the bacterial supernatant-synthesized AgNPs had a zeta potential of −20.3 mV (Figure 2e). For the citrate-reduced AgNPs, it was −21.6 mV (Figure 2f). The results indicated that the nanoparticles have good colloidal stability. The natural capping agents from the plant extract and bacterial supernatant likely contribute to this stability by preventing aggregation and maintaining a stable nanoparticle dispersion in the solution.

### 2.4. TEM Analysis

TEM provided detailed insights into the morphology and size of the synthesized AgNPs. TEM images revealed that the AgNPs synthesized using plant extract were predominantly spherical with an average size of 19.6 ± 5.8 nm, though some anisotropic shapes, such as triangles and rods, were also observed (Figure 2g). The bacterial supernatant-mediated AgNPs also displayed a predominantly spherical morphology with a slightly higher concentration of rods and triangles compared to the plant-derived AgNPs, with an average size of 23.3 ± 9.4 nm (Figure 2h). On the other hand, the citrate-reduced AgNPs were uniformly spherical, with an average size of 18.9 ± 2.1 nm (Figure 2i). Overall, TEM analysis confirmed that all three synthesis methods produced nanoparticles with comparable size ranges, with slight variations in morphology distribution depending on the synthesis route.

### 2.5. Elemental Analysis by EDX Spectroscopy

Elemental mapping and composition analysis using EDX spectroscopy showed a strong signal for silver, validating the successful synthesis of AgNPs by all three methods (Figure 2j–o).

### 2.6. Stability Analysis of AgNPs

**Figure 3:**
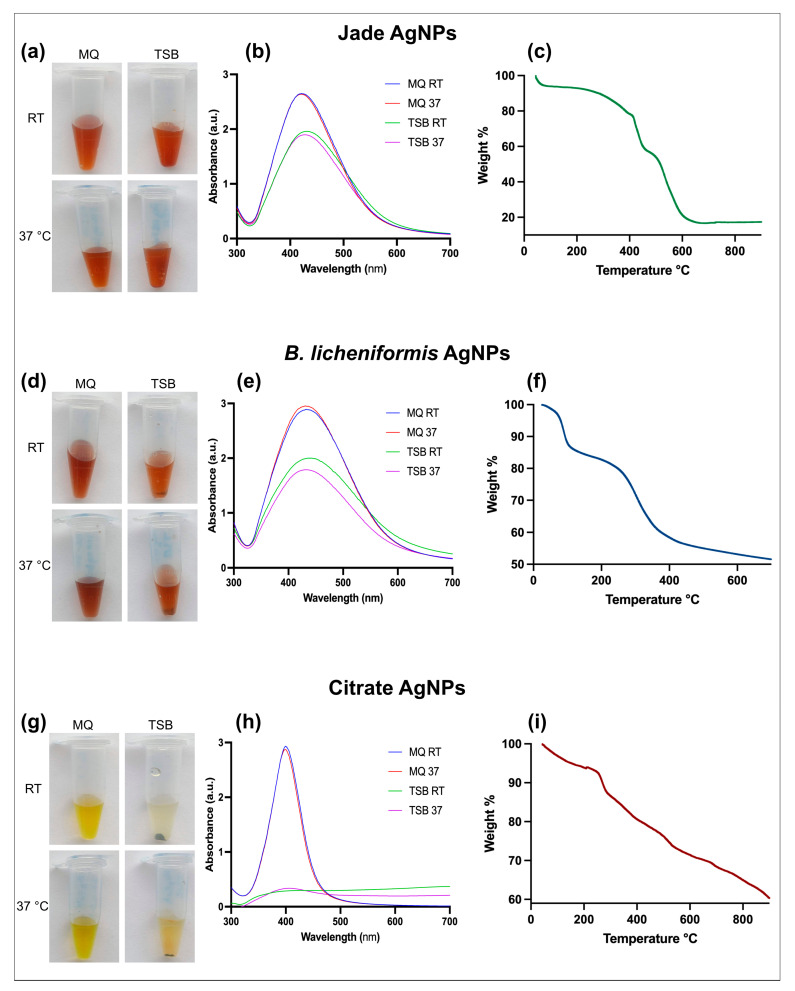
Visible analysis of AgNPs stability in MQ and TSB at room temperature and 37 °C (**a**) Jade AgNPs, (**d**) *B. licheniformis* AgNPs, (**g**) Citrate AgNPs. UV-vis spectra of AgNPs under various conditions (**b**) Jade AgNPs, (**e**) *B. licheniformis* AgNPs, (**h**) Citrate AgNPs. TGA analysis of (**c**) Jade AgNPs, (**f**) *B. licheniformis* AgNPs, and (**i**) Citrate AgNPs.

The stability of AgNPs was assessed after being stored for 15 days in water and TSB at both room temperature and 37 °C (Figure 3). AgNPs stored in water showed no visible signs of precipitation or colour change. This was confirmed by UV-Vis spectroscopy, which indicated that the nanoparticles remained stable during the storage period. However, stability differed for AgNPs stored in TSB. Plant extract and bacterial supernatant-synthesized AgNPs exhibited slight precipitation and small sediment formation at both room temperature and 37 °C. There was also a minor shift in the UV-Vis absorption spectrum, suggesting a mild degree of aggregation or structural change. In contrast, citrate-capped AgNPs showed significant instability when stored in TSB, with visible precipitation and notable sedimentation at both temperatures. UV-Vis analysis indicated a nearly complete loss of the characteristic absorption peak, signifying severe aggregation and breakdown of nanoparticle structure. In comparison, biologically synthesized AgNPs from plant extract and bacterial supernatant demonstrated superior stability with only slight precipitation and minor shifts in the UV-Vis spectra. These results indicate that biologically synthesized AgNPs offer better stability in complex media such as TSB, making them more suitable for long-term applications in biologically relevant environments.

The TGA of AgNPs synthesized using plant extract, bacterial supernatant, and citrate revealed distinct thermal degradation profiles (Figure 3c,f,i). For citrate-capped AgNPs, a minor weight loss below 150 °C was observed, likely due to the evaporation of adsorbed water and residual solvents. A more pronounced weight loss between 200 °C and 300 °C indicated the degradation of the organic citrate capping agent, with the derivative curve showing a peak in this range. After 600 °C, the weight loss stabilized, leaving a residual mass of approximately 60%, corresponding to the thermally stable silver core, which remained intact up to 900 °C. Similarly, plant extract synthesized AgNPs exhibited an initial minor weight loss below 150 °C, associated with the evaporation of moisture and volatile compounds, followed by a more substantial weight loss between 200 °C and 300 °C due to the decomposition of organic stabilizing agents. Additionally, a significant weight loss between 400 °C and 500 °C indicated the breakdown of more stable organic compounds, such as polysaccharides or proteins, leaving a residual mass of approximately 18%, representing the stable silver nanoparticles. In comparison, bacterial supernatant synthesized AgNPs showed a minor weight loss below 150 °C due to moisture loss, followed by a major degradation phase between 200 °C and 300 °C attributed to proteins and other organic stabilizers. The most significant weight loss occurred between 300 °C and 400 °C, marking the breakdown of more complex organic compounds like polysaccharides or lipids, leaving a residual mass of approximately 50%, representing the thermally stable AgNPs core. Together, these analyses highlight differences in the thermal stability and decomposition patterns between biologically and chemically synthesized AgNPs, with citrate-capped AgNPs showing higher residual mass, while plant extract and bacterial supernatant AgNPs demonstrated more complex organic decomposition.

### 2.7. FTIR Analysis

**Figure 4:**
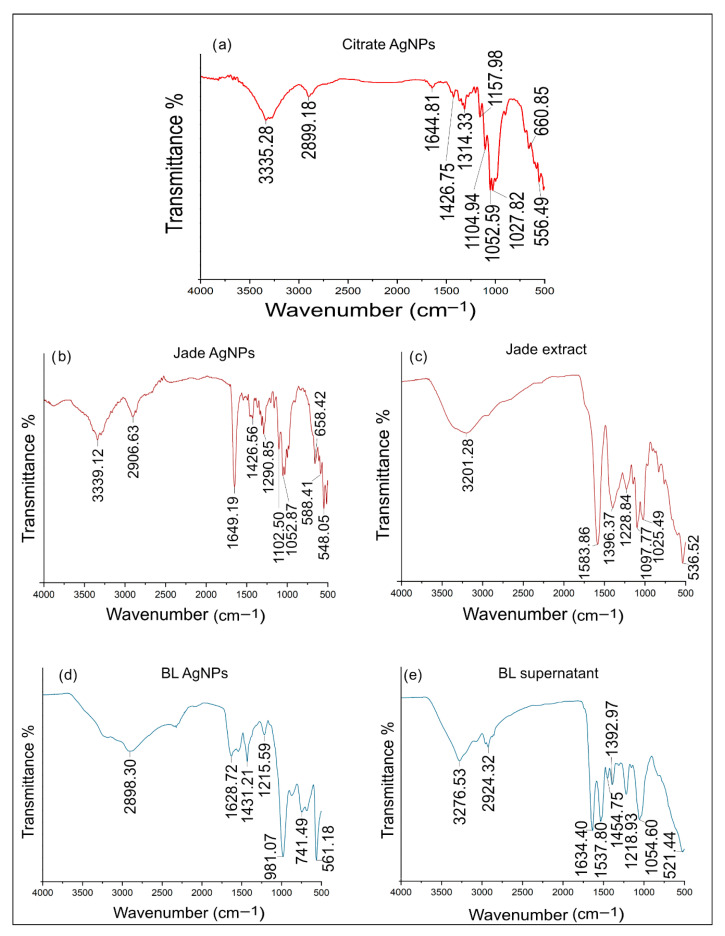
FTIR spectra of (**a**) Citrate AgNPs, (**b**) Jade AgNPs, (**c**) Jade extract, (**d**) *B. licheniformis* AgNPs, and (**e**) *B. licheniformis* supernatant.

FTIR was used to identify the functional groups responsible for the reduction and stabilization of AgNPs. FTIR analysis of AgNPs capped with citrate and tannic acid exhibited distinct peaks at 3335, 2899, 1644, 1426, 1314, 1157, 1104, 1052, 1027, 660, and 556 cm^−1^, indicating effective capping and stabilization by these organic molecules (Figure 4a) [25]. The broad peak at 3335 cm^−1^ corresponds to O-H stretching vibrations from hydroxyl groups in both citrate and tannic acid, suggesting hydrogen bonding interactions. The peak at 2899 cm^−1^ is attributed to C-H stretching in aromatic rings, particularly from tannic acid. The band at 1644 cm^−1^ corresponds to C=O stretching, indicative of carboxylate groups from citrate and possibly carbonyl groups in tannic acid. Peaks at 1426 and 1314 cm^−1^ relate to symmetric and asymmetric COO^−^ stretching, confirming the presence of citrate on the nanoparticle surface. The series of peaks between 1157 and 1027 cm^−1^ correspond to C-O stretching vibrations, derived from ester and phenolic groups in tannic acid. Finally, the peaks at 660 and 556 cm^−1^ are likely due to out-of-plane C-H bending in aromatic rings and Ag-O stretching, indicating strong interactions between the capping agents and the silver nanoparticle surface. Similarly, FTIR analysis of plant extract-synthesized silver nanoparticles revealed key peaks at 3339, 2906, 1649, 1426, 1290, 1102, 1052, 658, 588, and 548 cm^−1^, reflecting the contribution of various plant biomolecules to the reduction and stabilization of the nanoparticles (Figure 4b,c). The broad peak at 3339 cm^−1^ corresponds to O-H stretching, likely from hydroxyl groups, suggesting the involvement of polyphenols and other plant-based alcohols in nanoparticle capping. The peak at 2906 cm^−1^ is associated with C-H stretching, indicating the presence of aliphatic chains from plant lipids or proteins. The strong band at 1649 cm^−1^ corresponds to C=O stretching, characteristic of amides or carboxylates, indicating the role of proteins or organic acids in nanoparticle stabilization. Peaks at 1426 and 1290 cm^−1^ correspond to COO^−^ stretching and bending, confirming the presence of carboxylate groups from organic acids in the plant extract. The peaks at 1102 and 1052 cm^−1^ correspond to C-O stretching, likely from alcohols, esters, or glycosides. Finally, the peaks at 658, 588, and 548 cm^−1^ may be related to C-H out-of-plane bending or Ag-O stretching, reflecting strong interactions between plant-derived compounds and the nanoparticle surface [26]. Furthermore, FTIR analysis of AgNPs synthesized using bacterial supernatant revealed characteristic peaks at 2898, 1628, 1431, 1215, 981, 741, and 561 cm^−1^, indicating the involvement of various biomolecules in the reduction and stabilization of the nanoparticles (Figure 4d,e). The peak at 2898 cm^−1^ corresponds to C-H stretching vibrations, suggesting the presence of aliphatic chains, possibly from lipids or fatty acids in the bacterial metabolites. The band at 1628 cm^−1^ is associated with C=O stretching, characteristic of amide groups likely originating from proteins or peptides in the supernatant, which play a crucial role in capping the nanoparticles. The peak at 1431 cm^−1^ corresponds to COO^−^ symmetric stretching, indicating the involvement of carboxylate groups from organic acids or amino acids. The band at 1215 cm^−1^ can be attributed to C-N stretching vibrations, further supporting the presence of proteins or nucleic acids in the capping layer. The peaks at 981 and 741 cm^−1^ are related to C-H bending vibrations, possibly from aromatic residues or polysaccharides, reflecting contributions from bacterial cell wall components. Finally, the peak at 561 cm^−1^ is likely due to Ag-O stretching vibrations, indicating strong interactions between silver ions and biomolecules from the bacterial supernatant [27]. This spectrum highlights the complex capping environment provided by plant extracts and bacterial supernatants, where various functional groups from proteins, lipids, and other biomolecules contribute to the stability and functionality of the synthesized AgNPs.

### 2.8. Comparison of Antibacterial Activity

**Figure 5:**
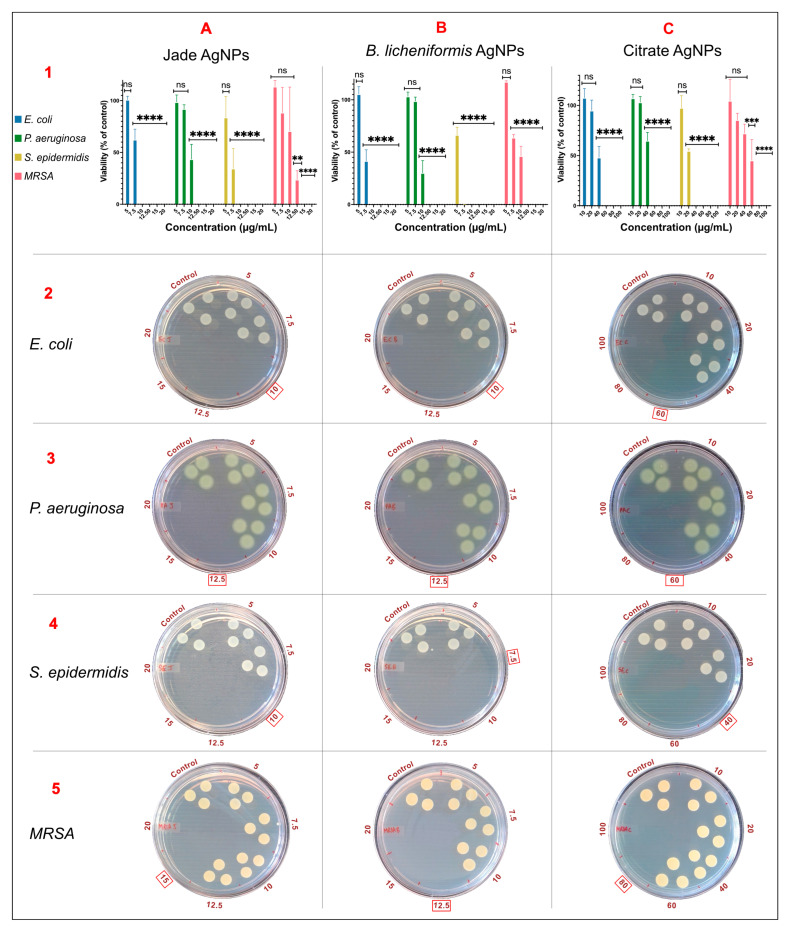
MBC values as determined by resazurin assay (row 1) and spot assay (row 2–5). Column (**A**) Jade AgNPs, (**B**) *B. licheniformis*, and (**C**) Citrate AgNPs. The red box marks the MBC values. Statistical significance is indicated as **** p < 0.0001, *** p < 0.001, ** p < 0.01, and ns = not significant (compared to control).

MIC and MBC values were determined to ascertain the bacteriostatic and bactericidal activity of biologically and chemically produced nanoparticles. The MIC values were established based on the visual inspection of wells, specifically the wells’ turbidity and the alamarBlue reagent’s color change from blue to pink, indicating bacterial growth. The MIC values for the Jade-synthesized AgNPs were as follows: 7.5 µg/mL for *E. coli*, 10 µg/mL for *P. aeruginosa*, 7.5 µg/mL for *S. epidermidis*, and 12.5 µg/mL for MRSA. Similarly, the AgNPs synthesized using bacterial supernatant exhibited MIC values of 7.5, 10, 7.5, and 10 µg/mL for *E. coli*, *P. aeruginosa*, *S. epidermidis*, and MRSA, respectively. In comparison, the citrate-reduced AgNPs demonstrated higher MIC values, with 40 µg/mL for *E. coli*, 60 µg/mL for *P. aeruginosa*, 20 µg/mL for *S. epidermidis*, and 60 µg/mL for MRSA. MBC values were determined based on an initial screening using fluorescence reading of the alamarBlue reagent post-incubation, followed by spotting 10 µl of the sample onto agar plates to determine bacterial viability. The MBC values for the plant extract-synthesized AgNPs were 10 µg/mL for *E. coli*, 12.5 µg/mL for *P. aeruginosa*, 10 µg/mL for *S. epidermidis*, and 15 µg/mL for MRSA (Figure 5A). The bacterial supernatant-synthesized AgNPs showed MBC values of 10 µg/mL for *E. coli*, 12.5 µg/mL for *P. aeruginosa*, 7.5 µg/mL for *S. epidermidis*, and 12.5 µg/mL for MRSA (Figure 5B). The citrate-reduced AgNPs, consistent with their MIC results, exhibited higher MBC values: 60 µg/mL for *E. coli*, 60 µg/mL for *P. aeruginosa*, 40 µg/mL for *S. epidermidis*, and 80 µg/mL for MRSA (Figure 5C). Comparing the MIC and MBC values across the different synthesis methods, green AgNPs required lower concentrations to achieve inhibitory and bactericidal effects. This shows their strong antimicrobial activity. The strong antimicrobial activity observed in the plant-based and bacterial AgNPs can also be explained by their surface chemistry and interaction with bacterial cells. The organic compounds present in biological extracts may facilitate stronger adhesion of AgNPs to cell membranes, which enhances membrane disruption and promotes ROS generation. Both effects accelerate cell death. In addition, AgNPs can release silver ions that bind to thiol-containing proteins and enzymes, interfering with respiration and DNA replication. The disruption of these essential pathways likely contributed to the lower MIC and MBC values observed for the green-synthesized nanoparticles. Their ability to penetrate and destabilize the biofilm matrix is also consistent with known AgNPs mechanisms, which include EPS disruption and increased membrane permeability within biofilm-associated cells. Although the AgNPs synthesized using the chemical method also demonstrated antimicrobial activity, their MIC and MBC values were generally higher, indicating a slightly reduced efficacy compared to the biologically synthesized nanoparticles. To compare the efficacy of all three nanoparticles in eradicating biofilms of *E. coli*, *P. aeruginosa*, *S. epidermidis*, *and MRSA*, several methods were employed.

**Figure 6:**
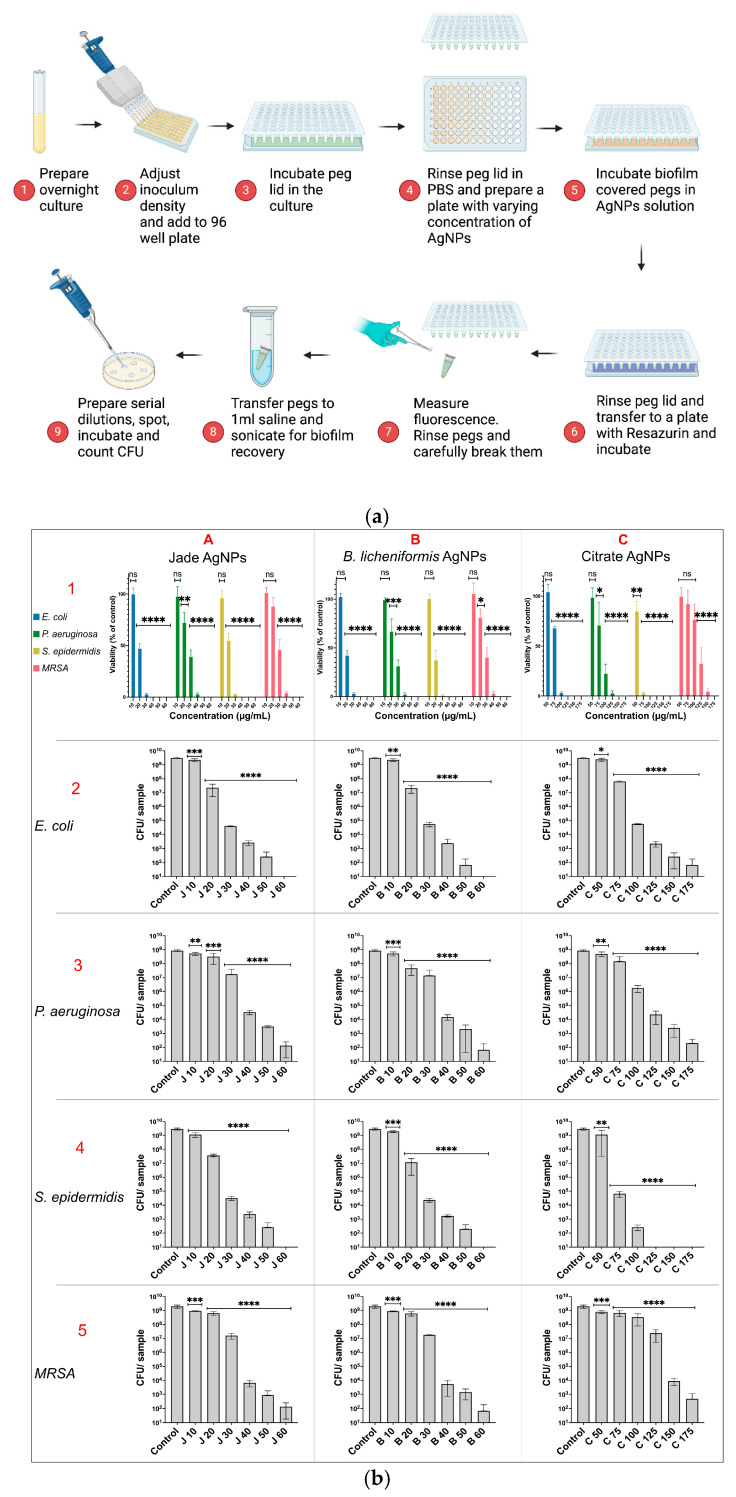
(**a**): Method for assessing biofilm eradication efficacy of the nanoparticles. (**b**): Effect of AgNPs on biofilm eradication as determined using resazurin assay (row 1) and CFU counting (row 2–5). Column (**A**) Jade AgNPs, (**B**) *B. licheniformis* AgNPs, and (**C**) Citrate AgNPs. AgNPs, and 125 µg/mL for citrate AgNPs. Complete eradication required higher concentrations: 50 µg/mL for both plant and bacterial AgNPs and 175 µg/mL for citrate AgNPs. Statistical significance is indicated as **** p < 0.0001, *** p < 0.001, ** p < 0.01, * p < 0.05.

Firstly, alamarBlue (resazurin), a viability indicator, was employed to measure metabolic activity within biofilms following treatment with each type of AgNPs (Figure 6b row 1). All three nanoparticles showed concentration-dependent eradication of the bacterial biofilms. At lower concentrations, the plant extract and bacterial supernatant-synthesized AgNPs exhibited the highest biofilm eradication efficacy, as indicated by a significant reduction in fluorescence intensity compared to untreated controls, whereas citrate AgNPs demonstrated the least biofilm eradication efficacy, with higher fluorescence intensity remaining, indicating residual metabolic activity within the biofilms. A significant reduction in viability was observed in *E. coli* biofilms treated with 20 µg/mL of plant and bacterial AgNPs with complete eradication at 40 µg/mL, whereas for citrate AgNPs, the values were 75 and 125 µg/mL, respectively. *P. aeruginosa* biofilms showed a similar pattern, with plant and bacterial AgNPs causing a significant reduction in viability at 20 µg/mL and a complete loss of viability at 50 µg/mL. Citrate AgNPs were less effective, requiring 75 µg/mL for significant reduction and 150 µg/mL for complete eradication. Interestingly, S. epidermidis biofilms were more susceptible to all three nanoparticles. Significant reductions in viability were observed at 20 µg/mL for both plant and bacterial AgNPs and at 50 µg/mL for citrate AgNPs. Complete eradication of S. epidermidis biofilms occurred at 40 µg/mL for both plant and bacterial AgNPs and at 100 µg/mL for citrate AgNPs. MRSA biofilms were the most resistant to nanoparticle treatment. Significant reductions in viability were observed at 30 µg/mL for plant AgNPs and 20 µg/mL for bacterial nanoparticles whereas, for citrate AgNPs, it was 125 µg/mL. Complete eradication of MRSA biofilms occurred at 50 µg/mL for both plant and bacterial AgNPs and at 175 µg/mL for citrate AgNPs.

**Figure 7:**
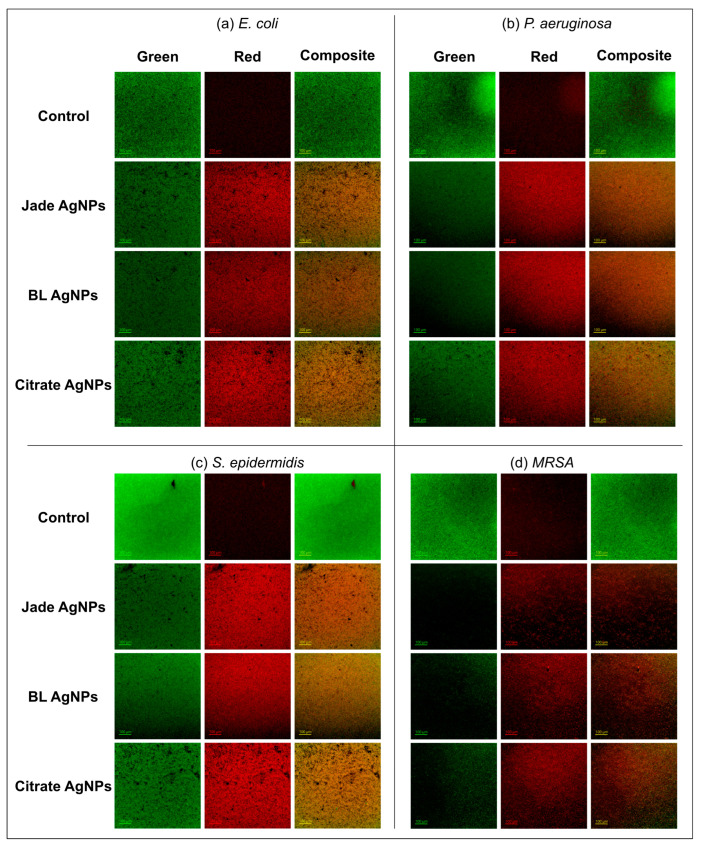
Live/Dead staining of biofilm samples post AgNPs treatment. (**a**) *E. coli*, (**b**) *P. aeruginosa*, (**c**) *S. epidermidis*, and (**d**) MRSA. Green—live/all cells, Red—dead cells. Scale bar: 100 µm.

To corroborate the resazurin assay results, Live/Dead staining was performed to visualize bacterial viability within biofilms after AgNPs treatment. At MBEC values for each AgNPs, a predominant red fluorescence (indicating dead cells) was observed across all tested bacterial strains, confirming their biofilm-killing capability (Figure 7). Though some green fluorescence was also observed in all the treated samples as the live dye, Syto9 stains both live and dead cells, and the intensity of green fluorescence is quenched in the presence of propidium iodide (red). The stacked images of AgNP-treated samples showed a distinct orange hue, which was not observed in control.

Furthermore, to quantify biofilm eradication, CFU counts were performed after biofilm treatment with different AgNPs. The results indicated a similar trend to that of the resazurin assay (Figure 6b rows 2–5). However, CFUs were still observed, despite multiple log reductions compared to the control, at concentrations where resazurin indicated complete biofilm eradication. This discrepancy could be attributed to the limit of detection, significant variation in cell density, and short incubation time with the dye [28].

**Figure 8:**
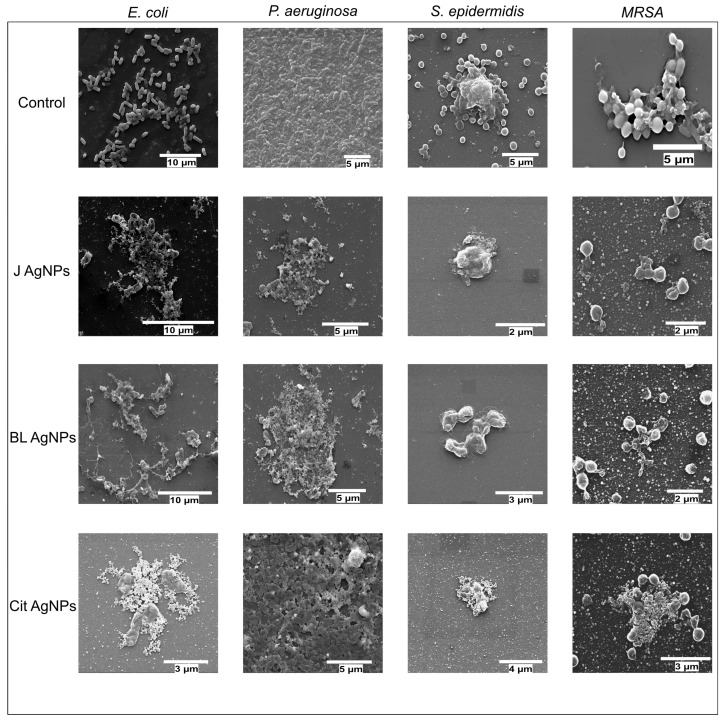
SEM micrographs of *E. coli*, *P. aeruginosa*, *S. epidermidis*, and MRSA biofilms with and without AgNPs treatment. Healthy and uniform biofilms can be seen in control samples while clear damage to the cell membrane and disruption of biofilms was observed with AgNPs treatments.

**Figure 9:**
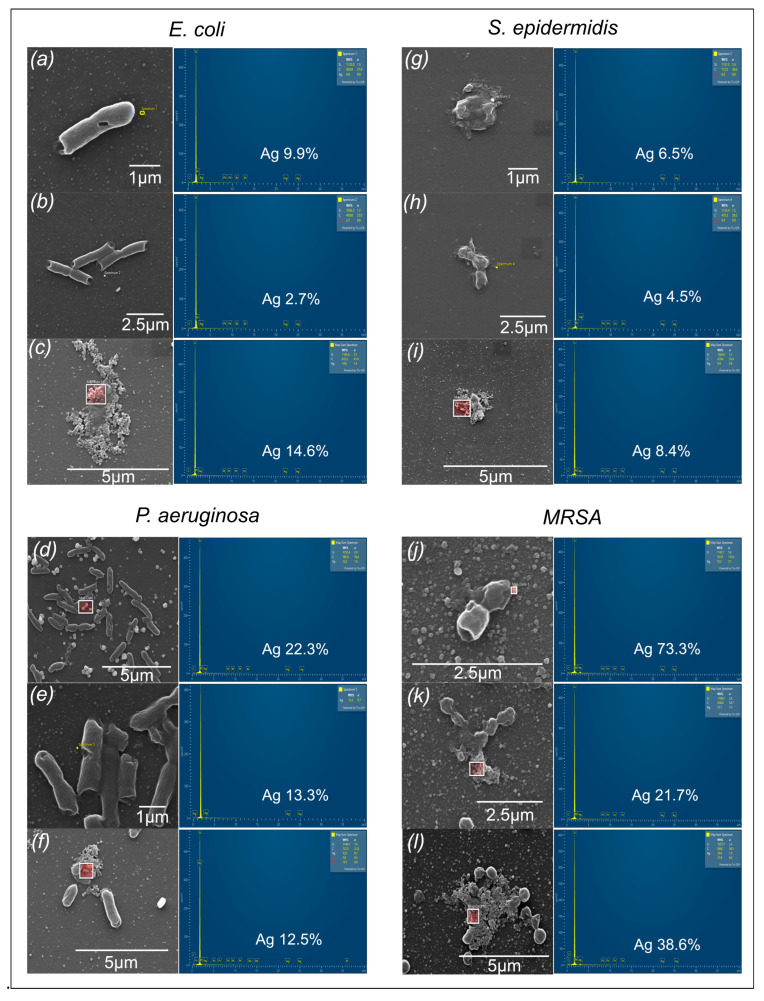
SEM micrographs and EDX spectra of treated biofilms of *E. coli:* (**a**) Jade AgNPs, (**b**) BL AgNPs, (**c**) citrate AgNPs; *P. aeruginosa*: (**d**) Jade AgNPs, (**e**) BL AgNPs, (**f**) citrate AgNPs; *S. epidermidis*: (**g**) Jade AgNPs, (**h**) BL AgNPs, (**i**) citrate AgNPs; and MRSA (**j**) Jade AgNPs, (**k**) BL AgNPs, (**l**) citrate AgNPs, confirming the presence of AgNPs in the samples.

Additionally, SEM was used to provide visual evidence of biofilm disruption and cell damage. SEM images of untreated biofilms revealed dense, well-structured biofilms for all bacterial strains. Post-treatment with AgNPs, SEM images showed significant disruption of the biofilm architecture, with sparse, fragmented bacterial cells with damaged membranes and a lack of extracellular polymeric substance (EPS) matrix, indicating effective biofilm eradication (Figure 8). Also, elemental mapping confirmed the presence of silver around the damaged cells, further supporting the biofilm eradication efficacy of the nanoparticles (Figure 9).

The results of these biofilm eradication experiments showed that biologically synthesized AgNPs exhibited the highest efficacy in eradicating biofilms across all tested bacterial strains at lower concentrations. This was evidenced by the significant reduction in biofilm viability as indicated by the resazurin assay, a higher proportion of dead cells in the live/dead staining, lower CFU counts, and clear visual evidence of biofilm disruption and cellular damage under SEM. While citrate-synthesized AgNPs also demonstrated considerable biofilm eradication efficacy, it was observed at higher concentrations compared to that of the biologically synthesized nanoparticles. These findings suggest that biofilm eradication efficacy varies among different synthesis methods of AgNPs, with bacteria and plant-synthesized AgNPs exhibiting the most potent biofilm eradication activity against the tested bacterial biofilms.

## 3. Discussion

This study provides a comprehensive evaluation of the antimicrobial and biofilm eradication efficacy of AgNPs synthesized through three distinct methods: plant extract-mediated synthesis, bacterial supernatant-mediated synthesis, and conventional chemical synthesis using citrate reduction. Our findings highlight the significant impact of the synthesis method on the physicochemical properties of AgNPs, which in turn influence their antimicrobial activity and biofilm eradication capabilities.

Green synthesis of nanoparticles has emerged as a sustainable and eco-friendly alternative to conventional chemical synthesis methods [29]. This approach leverages the natural reducing and capping agents present in plant and bacterial extracts to produce nanoparticles with distinct physicochemical properties. In our study, AgNPs were synthesized using Jade plant leaf extract and *B. licheniformis* bacterial supernatant, resulting in nanoparticles with distinct characteristics that contributed to their superior stability and antimicrobial efficacy. The use of plant extracts in nanoparticle synthesis offers several advantages. Plants are rich in a diverse array of phytochemicals, including flavonoids, terpenoids, alkaloids, and phenolic compounds, which act as both reducing and capping agents [30]. These biomolecules facilitate the reduction of silver ions to AgNPs and stabilize the nanoparticles by forming a protective organic layer on their surface. This capping layer is thought to enhance the biological activity of the nanoparticles by improving their interaction with microbial cells [31]. UV-Vis spectroscopy and TEM analyses revealed that AgNPs synthesized using jade extract had a moderately uniform size distribution with some anisotropic shapes, which may contribute to their enhanced antimicrobial properties. FTIR analysis further confirmed the presence of various functional groups, such as hydroxyl, carbonyl, and amine groups, which are known to play a role in stabilizing the nanoparticles and enhancing their interaction with bacterial cells. The DLS analysis showed that green-synthesized nanoparticles had larger hydrodynamic size compared to citrate AgNPs, which further confirmed the presence of thick corona layers surrounding the nanoparticles, thus providing them extra stability and enhancing their antibacterial activity. Notably, TEM images show that Jade AgNPs are surrounded by a dense organic corona, likely derived from plant biomolecules.

The plant extract-synthesized AgNPs consistently showed lower MIC and MBC values across all tested bacterial strains, including *E. coli*, *P. aeruginosa*, *S. epidermidis*, and MRSA. This suggests that biologically synthesized AgNPs are more potent in inhibiting and killing bacterial pathogens at lower concentrations. These findings are consistent with other studies that have demonstrated the effectiveness of plant-mediated synthesis of AgNPs. For instance, Wasilewska et al. reported the synthesis of AgNPs using extracts of various fruits and vegetables [32]. The antimicrobial activity of the AgNPs was tested against both Gram-positive and Gram-negative bacteria. It was observed that the size and potency of the nanoparticles varied with the type of extract used. This was attributed to the presence of bioactive phytochemicals in the extract, which not only reduce silver ions but also provides a biologically active capping layer that increases the nanoparticles’ interaction with microbial cells. Similarly, Radzikowska-Büchner et al. found that AgNPs synthesized using *Tanacetum vulgare* L. extract demonstrated potent antibacterial and antifungal activity, with the high content of polyphenols and proteins in the extract acting as efficient capping agents that enhance the antimicrobial properties of the nanoparticles [33].

Bacterial synthesis of AgNPs, also known as microbial synthesis, is another green approach that utilizes the metabolic processes of bacteria to reduce silver ions and stabilize the resulting nanoparticles. Bacteria like *B. licheniformis*, used in our study, produce a variety of bioactive compounds, including enzymes, proteins, and extracellular polysaccharides, which facilitate the synthesis and stabilization of AgNPs [34]. TEM analysis of the AgNPs synthesized using bacterial supernatant showed a predominantly spherical morphology with some anisotropic particles, while FTIR analysis indicated the presence of amide, carboxylate, and phosphate groups, suggesting that proteins and other bacterial metabolites played a significant role in capping and stabilizing the nanoparticles.

The bacterial supernatant-synthesized AgNPs also showed lower MIC and MBC values across the tested bacterial strains, demonstrating their efficacy at lower concentrations. These results align with previous research that has highlighted the effectiveness of microbial synthesis of nanoparticles. For example, Esmail et al. demonstrated that AgNPs synthesized using a newly isolated species of *Bacillus* (ROM6) exhibited strong antibacterial activity against a range of pathogens, including *S. aureus*, *E. coli*, *P. aeruginosa*, *and A. baumannii* [35]. The study suggested that bacterial proteins and enzymes played a crucial role in stabilizing the nanoparticles and enhancing their reactivity, contributing to their potent antimicrobial effects. In another study, Al-asbahi et al. reported that AgNPs synthesized using a combination of *Lactobacillus* sp. and *Bacillus* sp. showed significant antibacterial activity against multidrug-resistant strains of *S. aureus* and *P. aeruginosa* [27]. These results highlight the broad applicability of bacterial synthesis in generating potent antimicrobial agents.

Chemically synthesized AgNPs, such as those produced by citrate reduction, are recognized for their uniform size and precise synthesis control [21]. However, they often require higher concentrations to achieve antimicrobial effects comparable to their green-synthesized counterparts [36]. This disparity is likely due to the absence of biologically active surface coatings, which are naturally present in plant extract and bacterial supernatant used in green synthesis methods [37]. In our study, while chemically synthesized AgNPs were effective, they needed higher concentrations to match the bacteriostatic and bactericidal effects of the plant and bacterial AgNPs [38]. The smaller size and more consistent distribution of citrate-reduced AgNPs offer advantages in applications requiring strict nanoparticle size control.

However, their simpler surface chemistry limits their interaction with microbial cells. This may explain why they require higher concentrations for effective antimicrobial activity. This observation is consistent with previous studies. For instance, Feng et al. reported that while citrate-reduced AgNPs exhibited antimicrobial activity, they were less effective at lower concentrations compared to AgNPs synthesized using glycyrrhizin [39]. Similarly, in a study by Borah et al., the antimicrobial activity of AgNPs produced using *O. sanctum* leaf extract was compared to that of citrate AgNPs against multidrug-resistant bacteria [40]. The researchers found that the biological AgNPs were more effective than the chemically produced nanoparticles. The study suggested that the presence of alkaloids in the extract enhanced the antimicrobial activity of the AgNPs. This indicates that the simpler surface chemistry of chemically synthesized nanoparticles might reduce their effectiveness against bacterial cells.

The results from the biofilm eradication assay further underscore the superior efficacy of plant extract and bacterial supernatant-synthesized AgNPs. In comparison to the citrate AgNPs, at lower concentrations, green AgNPs demonstrated the highest efficacy in eradicating biofilms across all tested bacterial strains, as evidenced by significant reductions in metabolic activity (resazurin assay), increased dead cell proportions (Live/Dead staining), lower CFU counts, and clear visual disruption of biofilm architecture (SEM analysis). These results suggest that the complex surface chemistry of biologically synthesized AgNPs enhances their ability to penetrate and disrupt the EPS matrix of biofilms, leading to more effective eradication of biofilm-embedded bacteria. On the other hand, the citrate-reduced AgNPs, while still effective, required higher concentrations to achieve similar levels of biofilm eradication. This could be due to the simpler surface chemistry of citrate-capped nanoparticles, which may limit their interaction with the biofilm matrix and the bacterial cells within it. Verduzco-Chavira et al. (2023) also observed that biologically produced AgNPs using *Jacaranda mimosifolia* flower extract inhibited biofilm formation more effectively than chemically synthesized AgNPs, despite requiring lower concentrations, highlighting the advantages of green synthesis [41]. Similarly, in a study by Oliver et al., the antimicrobial activity of citrate-capped AgNPs was compared to that of green AgNPs synthesized using catechin, a polyphenol [42]. The study found that the biologically produced AgNPs caused a 99% reduction in biofilm at a concentration of 5 µg/mL, whereas no reduction was observed when using citrate AgNPs at the same concentration. Furthermore, it was discovered that the uptake of catechin AgNPs was over 20 times higher than citrate AgNPs. The conclusion drawn was that using polycat as a capping agent significantly increased the contact between AgNPs and bacteria cells, thereby improving antibacterial efficacy. The size and shape of nanoparticles are critical factors influencing their biological activity. In our study, AgNPs synthesized using *C. ovata* and *B. licheniformis* extract were found to have a slightly broader size distribution and some anisotropic shapes (e.g., rods and triangles), which may enhance the surface area available for interaction with microbial cells, leading to greater antimicrobial efficacy. This finding is supported by research from Kim et al. and Thammawithan et al., who reported that the anisotropic shapes of AgNPs contributed to their enhanced antimicrobial activity against a wide range of bacterial pathogens [43,44]. Similarly, Lima et al. reported that the antimicrobial activity of AgNPs synthesized using *Ilex paraguariensis* was dependent on the morphology of the nanoparticles [45].

Another significant advantage of green-synthesized AgNPs is their biocompatibility. The use of natural extracts as reducing and capping agents reduces the potential for toxicity, making these nanoparticles more suitable for medical and pharmaceutical applications. Studies have shown that green AgNPs exhibit lower cytotoxicity toward mammalian cells compared to chemically synthesized nanoparticles, making them safer for use in antimicrobial therapies [46]. Veeragoni et al. demonstrated that green AgNPs using *Padina tetrastromatica* showed lower genotoxicity as compared to chemically synthesized AgNPs both in vitro and in vivo, highlighting their potential for safe therapeutic applications [47]. Similarly, a recent study by Ongtanasup et al. reported the synthesis of AgNPs from *Zingiber officinale* extract exhibiting biocompatibility at concentrations below 17.5 µg/mL and also showed anti-inflammatory properties [48]. Overall, these results confirm the effectiveness of AgNPs synthesized by all three methods. Bacterial AgNPs stand out as the most potent, closely followed by plant-based AgNPs against both Gram-negative and Gram-positive bacteria, including MRSA. The findings of this study have significant implications for the development of AgNPs-based antimicrobial therapies. The superior antimicrobial and biofilm eradication efficacy of biologically synthesized AgNPs suggests that green synthesis methods could provide a viable and sustainable alternative to conventional chemical methods. The use of plant extract and bacterial supernatant not only reduces the environmental and health risks associated with chemical synthesis but also enhances the biological activity of the resulting nanoparticles. The potent activity of biologically synthesized AgNPs against MRSA highlights their potential as effective agents in the fight against antimicrobial resistance. The ability of these nanoparticles to eradicate biofilms further supports their use in treating chronic and hard-to-treat infections where biofilms are a major complicating factor [49].

## 4. Materials and Methods

Silver nitrate (AgNO_3_), trisodium citrate (Na_3_C_6_H_5_O_7_), and tannic acid (C_76_H_52_O_46_) tryptic soy broth (TSB), Mueller Hinton (MH) medium, Dulbecco’s phosphate-buffered saline (DPBS) were purchased from Sigma Aldrich (Merck) (Søborg, Denmark). AlamarBlue™ HS Cell Viability Reagent, LIVE/DEAD™ *Bac*Light™ Bacterial Viability Kit (L13152), and Nunc™ Immuno TSP Lids were purchased from ThermoFisher Scientific (Roskilde, Denmark). Glutaraldehyde, 2.5% In 0.1 M Sodium Cacodylate Buffer was purchased from Axlab A/S (Vedbæk, Denmark).

### 4.1. Synthesis of AgNPs

#### 4.1.1. Green Synthesis of AgNPs from Jade Plant

Jade plant was purchased from a local florist. The leaves were washed with distilled water to remove contaminants and air-dried. Twenty-five grams of the leaves were coarsely cut and added to a sterile flask containing 100 mL distilled water and autoclaved for 15 min at 121 °C. After extraction of the phytochemicals, the mixture was filtered through Whatman filter paper, grade 1, and centrifuged at 8000× *g* for 5 min. For nanoparticle synthesis, different concentrations of AgNO_3_ and the leaf extract were mixed and stirred at room temperature (RT). Nanoparticle synthesis was confirmed by a color change in the reaction mixture to dark brown, followed by spectral analysis. The nanoparticles were centrifuged at 5000× *g* for 3 min to remove bigger particles, followed by centrifugation and washing thrice with distilled water at 45,000× *g* for 30 min to remove unreacted silver salt and leaf extract. The pellet was resuspended in Milli-Q water and stored in a refrigerator until further analysis.

#### 4.1.2. Green Synthesis of AgNPs from *B. licheniformis*

A previously isolated strain of *B. licheniformis* (5A36) was used for the synthesis. Briefly, the isolate was inoculated into 100 mL of TSB followed by incubation at 37 °C for 24 h at 150 rpm. The bacterial cells were separated by centrifugation at 8000× *g* for 5 min. This cell-free supernatant was used for nanoparticle synthesis. For optimization, different concentrations of AgNO_3_ were added to the cell-free supernatant and incubated in a rotary mixer at RT. A control comprising sterile TSB and AgNO_3_ was also added. The synthesis of nanoparticle was visually confirmed by colour change and confirmed with spectral analysis. Like Jade AgNPs, the nanoparticles were centrifuged at 5000× *g* for 3 min to remove bigger particles, followed by centrifugation and washing thrice with distilled water at 45,000× *g* for 30 min to remove unreacted silver salt. The pellet was resuspended in Milli-Q water and stored at 4 °C for further analysis.

#### 4.1.3. Chemical Synthesis—Citrate Reduction

The chemical synthesis of AgNPs was carried out using a previously described method with slight modifications [21]. Briefly, 200 mL of distilled water containing five mM trisodium citrate and 0.025 mM tannic acid was brought to a boil, and 1 mL of AgNO_3_ (25 mM) was added to the solution under stirring. After the appearance of yellow color, the flask was cooled, followed by the addition of trisodium citrate, tannic acid, and AgNO_3_ in succession. After initial centrifugation at 5000× *g* to remove bigger particles, the nanoparticles were washed thrice at 45,000× *g* to remove excess chemicals, and the pellet was resuspended in Milli-Q water and stored in a refrigerator for further analysis.

### 4.2. Characterization of AgNPs

#### 4.2.1. UV-Vis

The synthesis of AgNPs was first confirmed by visual inspection, followed by acquiring a spectrum using a UV-Vis spectrophotometer. A small aliquot of the nanoparticle solution was diluted and transferred to a quartz cuvette for analysis. The UV-Vis absorption spectrum was recorded over a wavelength range of 300–700 nm at RT. The baseline was corrected using deionized water as a blank before each measurement. The UV-vis spectrum was also used for the optimization of synthesis parameters for the biologically produced nanoparticles.

#### 4.2.2. Dynamic Light Scattering (DLS)

For DLS analysis, the original AgNPs suspension was diluted with Milli-Q water to ensure optimal particle concentration for accurate measurement. A 1 mL aliquot of the diluted suspension was analyzed using a Malvern Zetasizer Nano ZS90 (Malvern Panalytical, (Malvern, UK) to determine the hydrodynamic diameter and polydispersity index (PDI) of the nanoparticles. The zeta potential of the AgNPs was measured using a folded capillary zeta cell, also provided by Malvern Panalytical (Malvern, UK), to assess the stability of the nanoparticles in suspension.

#### 4.2.3. Stability Analysis

The stability of silver nanoparticles (AgNPs) was evaluated after a 15-day period by visually examining the samples for any changes in colour or precipitation. In addition, UV-Vis spectroscopy was employed to observe any shifts in the absorption spectra. The stability was tested in MQ water and TSB at both room temperature and 37 °C to identify any changes in the nanoparticles’ stability. Additionally, the thermal stability of citrate, Jade, and *B. licheniformis* AgNPs was assessed using thermogravimetric analysis (TGA) on a Discovery TGA, TA Instruments (New Castle DE, USA). The AgNPs suspensions were heated at a rate of 10 °C/min from ambient temperature to 700/900 °C.

#### 4.2.4. Inductively Coupled Plasma-Mass Spectrometry (ICP-MS)

For the determination of AgNPs concentration, ICP-MS was employed. The original AgNPs suspension was digested using nitric acid (HNO_3_) to dissolve the nanoparticles into ionic form. The resulting solution was then diluted to bring the silver concentration within the optimal detection range of the instrument. A 1 mL aliquot of the digested and diluted sample was analyzed using an ICP-MS instrument (Agilent 7850 ICP-MS, Agilent Technologies, Glostrup, Denmark)). The concentration of silver ions in the sample was determined by comparing the measured signal to a calibration curve generated using known silver standards.

#### 4.2.5. Fourier Transform-Infrared Spectroscopy (FTIR)

To identify the biomolecules and functional groups involved in the reduction and capping/stabilization of the AgNPs, FTIR spectroscopy was employed. The AgNPs suspensions were analyzed using a Nicolet iS50 spectrometer (Thermo Fisher Scientific, Waltham, MA, USA) in the spectral range of 500–4000 cm^−1^. The resulting FTIR spectra were plotted as transmittance (%) against wavenumber (cm^−1^).

#### 4.2.6. Transmission Electron Microscopy (TEM)

AgNPs were examined using TEM to obtain detailed images of their size, shape, and distribution. The samples were prepared by placing a drop of the diluted nanoparticle suspension onto a carbon-coated copper grid, followed by air drying at RT. TEM analysis was carried out using a FEI Tecnai T20 G2 (FEI Company, Hillsboro, OR, USA) operated at an accelerating voltage of 200 kV. High-resolution images were captured to assess the nanoparticles’ morphological characteristics, and the data were used to measure the particle size distribution and analyze the surface structure in detail.

#### 4.2.7. Elemental Mapping Using Energy Dispersive X-Ray (EDX)

For elemental mapping, a drop of the AgNPs suspension was placed onto a diced silicon wafer affixed to an aluminum stub. The sample was allowed to dry overnight and analyzed using a Quanta FEG 200 ESEM (FEI Company, Hillsboro, OR, USA) equipped with an Oxford X Max EDS detector (Oxford Instruments, High Wycombe, UK). Elemental analysis was conducted by focusing on areas with high concentrations of nanoparticles, allowing for the confirmation of silver content.

### 4.3. Antibacterial Activity of AgNPs

#### 4.3.1. Bacterial Strains

The antimicrobial activity of AgNPs was tested against four pathogens, including two types of Gram-positive bacteria (*Staphylococcus epidermidis* ATCC 35984 and Methicillin-resistant *Staphylococcus aureus* (MRSA) USA300_FPR3757) and two types of Gram-negative bacteria (*Escherichia coli* UTI 89 and *Pseudomonas aeruginosa* PAO1). LB medium was used for growing *E. coli* and *P. aeruginosa*, while TSB was used for *S. epidermidis* and *S. aureus*.

#### 4.3.2. Minimum Inhibitory Concentration (MIC) and Minimum Bactericidal Concentration (MBC)

The MIC and MBC of the AgNPs were determined using the broth microdilution method. Bacterial strains were grown overnight in their respective media at 37 °C. Varying dilutions of AgNPs (plant, bacteria, and citrate) were prepared in a 96-well microtiter plate. Each well was inoculated with approximately 5 × 10^5^ CFU/mL of the bacteria diluted in MH broth. The plates were incubated at 37 °C for 20 h. The MIC was defined as the lowest concentration of AgNPs that visibly inhibited bacterial growth. To determine the MBC, alamarBlue HS cell viability reagent was added to the wells and incubated for 1 h at 37 °C. After incubation with the dye, fluorescence was measured using an excitation wavelength of 560 nm and an emission wavelength of 590 nm. Additionally, 10 µL of samples from the wells were spotted on agar and incubated. The MBC was defined as the lowest concentration of AgNPs that resulted in a 99.9% reduction in the initial bacterial count, indicating bactericidal activity.

#### 4.3.3. Biofilm Eradication Assay

To assess the biofilm eradication efficacy of AgNPs, minimum biofilm eradication concentration (MBEC) assay was performed as described earlier with slight modifications [50]. Briefly, bacterial strains were grown overnight in their respective media at 37 °C. After overnight incubation, the inoculum density was adjusted to 1 × 10^7^ CFU/mL. Using a multichannel pipette, 150 µL of the adjusted inoculum was added to a 96-well microtiter plate, avoiding the outer wells. 150 µL water was added to the exterior wells (columns 1, 12 and rows A and H). Nunc immuno TSP lid was placed on the plate and was incubated at 37 °C in a humidified environment under shaking at 100 RPM. After 24 h of biofilm growth, the OD of all the wells was measured to ensure uniform growth. The peg lid was washed twice to remove dead and planktonic cells by transferring it to a microtiter plate containing 200 µl PBS. The peg lid was then transferred to a fresh microtiter plate containing 200 µL of varying concentrations of AgNPs and incubated at 37 °C for 20 h in a humidified environment under static conditions. After incubation with AgNPs, the pegs were washed twice with PBS, as mentioned above. To determine the viability of biofilm, the peg lid was transferred to a microtiter plate containing alamarBlue HS cell viability reagent and incubated at 37 °C for 1 h. Fluorescence was measured using an excitation wavelength of 560 nm and an emission wavelength of 590 nm. For CFU enumeration, the pegs were carefully broken and placed in a microcentrifuge tube containing 1mL of saline and sonicated for biofilm recovery. Tenfold serial dilutions of the cell suspension were performed, and 5 µL of the dilutions were spotted on an agar plate and incubated overnight at 37 °C. The number of colonies was counted, and CFU/mL for each well was determined.

#### 4.3.4. Live/Dead Staining

To visualize the fraction of live and dead bacteria, biofilms were grown in a 96-well microtiter plate under the same conditions as described above and treated with AgNPs. After treatment, cells were stained using LIVE/DEAD™ *Bac*Light™ Bacterial Viability Kit (L13152) as per the manufacturer’s protocol and imaged using ImageXpress^®^ Pico Automated Cell Imaging System (Molecular Devices, Berkshire, UK) along with their proprietary software (CellReporterXpress, version 2.9.4).

#### 4.3.5. Scanning Electron Microscopy (SEM) of Biofilms

SEM was employed to visualize the damage caused to cells in the biofilm. A 5 mm^2^ diced silicon wafer was placed inside the well of a 48-well plate, and biofilm was grown and treated as described above. After treatment, the silicon wafer was washed with PBS, and the biofilm was fixed with 2.5% glutaraldehyde for 2 h at RT and protected from light. After fixation, the wafer was washed with PBS to remove excess fixative and then dehydrated with a graded series of ethanol concentrations (40–90%) for 15 min each and transferred to absolute ethanol and left overnight in the refrigerator. The next morning, wafers were fixed on an aluminium stub using an adhesive carbon tab and left to air dry. The samples were sputter coated with platinum and imaged using a Quanta FEG 200 ESEM (FEI Company, Hillsboro, OR, USA) equipped with an Oxford X Max EDS detector (Oxford Instruments, High Wycombe, UK).

## 5. Conclusions

This study demonstrates that the method of synthesis plays a critical role in determining the antimicrobial efficacy and biofilm eradication potential of AgNPs. Biologically synthesized AgNPs, particularly those derived from plant extract and bacterial supernatant, exhibit superior antimicrobial properties compared to chemically synthesized AgNPs. These findings support the use of green synthesis methods for producing AgNPs with enhanced biological activities, offering promising applications in medical and pharmaceutical fields. Future research should explore the scalability of these green synthesis methods and their potential integration into clinical practice for combating antimicrobial resistance. Additionally, further studies should investigate the long-term stability, safety, and efficacy of green-synthesized AgNPs in vivo, as well as their potential synergistic effects when combined with existing antimicrobial therapies. Such research could pave the way for the development of next-generation antimicrobial agents that are both effective and environmentally sustainable.

## Data Availability

All data is provided in the paper; on request raw data will be available.
